# Screening for Anti-HMGCR Antibodies in a Large Single Myositis Center Reveals Infrequent Exposure to Statins and Diversiform Presentation of the Disease

**DOI:** 10.3389/fimmu.2022.866701

**Published:** 2022-05-04

**Authors:** Piotr Szczesny, Simone Barsotti, Inger Nennesmo, Olof Danielsson, Maryam Dastmalchi

**Affiliations:** ^1^ Department of Rheumatology, National Institute of Geriatrics, Rheumatology and Rehabilitation, Warsaw, Poland; ^2^ Rheumatology Unit, Department of Clinical and Experimental Medicine, University of Pisa, Pisa, Italy; ^3^ Rheumatology Outpatient Clinic, Livorno Hospital, Livorno, Italy; ^4^ Department of Laboratory Medicine Karolinska Institutet, Stockholm, Sweden; ^5^ Division of Neurology, Department of Biomedical and Clinical Sciences, Faculty of Medicine and Health Sciences, Linkoping University, Linkoping, Sweden; ^6^ Division of Rheumatology, Department of Medicine, Karolinska Institutet, Solna, Sweden; ^7^ Division of Rheumatology, Karolinska University Hospital, Stockholm, Sweden; ^8^ Center for Molecular Medicine, Karolinska Institutet, Stockholm, Sweden

**Keywords:** immune-mediated necrotizing myopathy (IMNM), anti-HMGCR, statin, dermatomyositis, idiopathic inflammatory myopathy

## Abstract

**Background:**

The objective of this study is to assess the frequency of autoantibodies against 3-hydroxy-3-methyl-glutaryl-coenzyme A reductase (HMGCR) in a single center myositis cohort and to analyze associations with statin exposure, clinical features, and outcome of disease course.

**Methods:**

A total of 312 patients with idiopathic inflammatory myopathies (IIMs) followed at the rheumatology clinic, Karolinska University Hospital, were identified in the Euromyositis registry between 1988 and 2014 and were classified according to the 2017 European Alliance of Associations for Rheumatology/American College of Rheumatology (EULAR/ACR) criteria. Available serum samples were analyzed for anti-HMGCR autoantibodies by ELISA. Positive sera were confirmed by immunoprecipitation. Clinical data were extracted from Euromyositis registry and medical records. Muscle samples were examined by two pathologists blinded to the subjects’ autoantibody status.

**Results:**

Of 312 patients, 13 (4.3%) were positive for anti-HMGCR. Two of the 13 (15%) anti-HMGCR–positive patients had histories of statin use versus 12 (4.2%) in the anti-HMGCR–negative group. In the anti-HMGCR–positive group, five (38%) had a clinical phenotype compatible with dermatomyositis. Muscle biopsies of patients with HMGCR autoantibodies showed findings consistent with immune-mediated necrotizing myopathy in all cases except for one. Five (38%) patients required treatment with intravenous immunoglobulin compared to seven (2.3%) without this antibody. At the last visit, seven patients had chronic, active disease course, and five of 13 patients were in remission, including three without treatment.

**Conclusions:**

Patients with IIM related to anti-HMGCR autoantibodies may present with a wide range of symptoms, more than previously anticipated. When a broad approach to screening for these antibodies is applied, only a minority of patients was found to have previous statin exposure. The results of this study justify the addition of anti-HMGCR autoantibodies to routine diagnostic procedures in patients with myositis.

## Introduction

Immune-mediated necrotizing myopathy (IMNM) is currently characterized as one of the most severe subtypes of idiopathic inflammatory myopathies (IIMs). It is known for pronounced proximal muscle weakness, increased serum levels of creatine kinase (CK), often widespread necrosis of muscle fibers on muscle biopsy with no or only mild infiltration of inflammatory cells, and high prevalence of life-threatening dysphagia with paucity of other extramuscular symptoms. As in other autoimmune myopathies, serology plays an important role in stating the diagnosis and predicting course of the disease. There are currently two known myositis-specific autoantibodies (MSAs) associated with IMNM: anti-SRP (signal recognition particle) and anti-HMGCR (3-hydroxy-3-methylglutaryl-coenzyme A reductase) (1). Statins, the main inhibitors of HMGCR, have been associated to the latter; however, the direct linkage has not been unequivocally proven.

Recently, pathogenicity of anti-HMGCR antibodies was demonstrated (2–4). When the antibodies were transferred from patients with positive IMNM to mice genetically modified with the human complement system, they were able to induce a similar disease and formation of C5b-9 (membrane attack complex, MAC) in the cellular membrane. *In vitro*, their presence produced muscle fiber atrophy. They also correlated with disease activity in individual patients (2). On the basis of these observations, clinical trials have already begun with the intention to selectively treat IMNM patients (e.g., zilucopan, a C5 inhibitor).

Because of low availability, testing for anti-HMGCR was rarely performed only in the most severe cases and without extra-muscular involvement. This selection bias resulted in many previous reports, suggesting that anti-HMGCR–related myopathy is an exclusively muscular disease with acute onset and unfavorable prognosis.

On the other hand, in these initial reports, only 44%–63% of IMNM patients with these antibodies had known history of statin exposure (3). There are reports of anti-HMGCR–related myopathy occurring in childhood (4, 5), whereby it is unlikely that lipid-lowering agents were prescribed. Therefore, with close examination of the literature, direct association with statin exposure becomes less evident. Moreover, the exclusive association between anti-HMGCR autoantibodies and the clinical subgroup IMNM has been questioned. There is a rising number of positive anti-HMGCR autoantibody cases reported with dermatomyositis (DM), other inflammatory diseases, or even with asymptomatic hyper-CK-emia (2, 3).

Our hypothesis was that anti-HMGCR–related IIM, contrary to the common paradigm, does not always present as the IMNM phenotype nor in association to statin exposure. This study was intended to screen a comprehensively characterized cohort of patients with IIM for frequency of anti-HMGCR autoantibodies and investigate the association with clinical manifestations, disease course, and previous exposure to statins.

## Methods

This is a single center retrospective study from rheumatology clinic at Karolinska University Hospital, Stockholm, Sweden. Subjects with a diagnosis of IIM between 1988 and 2014 were enrolled to ensure prospective follow-up. All patients were confirmed to have IIM, all retrospectively fulfilled the 2017 ACR/EULAR classification criteria for IIM (6). The most recent European Neuromuscular Centre (ENMC) criteria for IMNM were applied to the patients positive for anti-HMGCR autoantibodies (1). At the first clinic visit and after signing informed consent for study participation, blood samples were drawn and stored in −80°C freezer for later analysis. The study was approved by the Regional Ethics committee in Stockholm.

Information on demographic, clinical, and treatment data was retrieved from the international electronic myositis registry, Euromyositis, and patient’s records. Disease activity has been recorded in the form of the Core Set Measures (7) in Euromyositis prospectively at visits since 1993. As no approved definition of remission in IIM exists, disease activity was assessed by an experienced clinician based on patient’s records, physical examination, and change in the Core Set Measures as either high disease activity, chronic active disease, low disease activity, remission, or drug free remission.

All patients were screened for interstitial lung disease (ILD) with high-resolution computed tomography and pulmonary function tests: spirometry, plethysmography, and diffusing capacity for carbon monoxide. Dysphagia was confirmed by radiology with barium sulphate contrast. Arthritis, Raynaud’s phenomenon, and skin lesions were confirmed on physical examination by an experienced physician. Heart involvement was regarded at investigators discretion as any cardiac abnormality resulting from disease activity after other causes were excluded. All patients were screened for MSA by EUROIMMUNE Line blot assay and ELISA for anti-FHL1.

Anti-HMGCR autoantibodies were kindly analyzed by Dr. Andrew Mammen, Muscle Disease Unit, National Institutes of Health, USA. Serum samples from all patients (n = 312) were screened for anti-HMGCR autoantibodies by enzyme-linked immunosorbent assay (ELISA) and confirmed by immunoprecipitation of *in vitro* transcribed and translated HMGCR protein (IP-IVTT). Cutoff for positivity in ELISA was set as 0.35 arbitrary units (AU)/ml. Strong or weak positivity was determined by IP-IVTT strength of band.

Patient’s archived biopsies were all examined by two experienced muscle pathologists, who were blinded to autoantibody status (I. Nennesmo and O. Danielsson). Staining of biopsies was performed according to a standard myositis protocol. The following features were evaluated: necrotic myofibers, regenerating myofibers, inflammatory cell infiltrates, scattered inflammatory cells, perimysial infiltration, centralized nuclei, and major histocompatibility complex (MHC) class I upregulation. For all pathology features, a semi-quantitative scale was used: absent (“0”), mild (“1+”), moderate (“2+”), and pronounced (“3+”). Findings like endomysial infiltration, perifascicular atrophy, atrophic fibers, fiber size variation, and increased amount of adipose tissue were also examined.

Blood samples for genetic assessment have been stored and analyzed upon separate informed consent. Human leukocyte antigen (HLA)-DRB1 genotyping was performed by Olerup SSP DR low-resolution kit.

## Results

Of 312 patients enrolled 13 (4.3%) patients were positive for anti-HMGCR autoantibodies. All were Caucasian. All fulfilled the 2016 ENMC criteria for IMNM or anti-HMGCR myopathy (1). Median age at diagnosis of IIM in patients with anti-HMGCR autoantibodies was 52 years (range 34–75 years). Of the 13 anti-HMGCR–positive patients, 11 were women (92.3%) compared to 61% in the HMGCR-negative group.

### Statin Exposure

Two of 13 patients (15%) had been exposed to statins prior to disease onset—a 70-year-old woman and a 75-year-old man—had both been exposed to simvastatin. Both were classified as polymyositis according to the ACR/EULAR 2017 criteria. Among the 299 patients who were negative for anti-HMGCR antibodies, 12 (4.2%) had been exposed to statins prior to onset of disease. For 11 patients, the history of previous statin intake remained uncertain (**Table 1**).

**Table 1:** Comparison between anti-HMGCR–positive and anti-HMGCR–negative groups.

	Anti-HMGCR–positive IIM	Anti-HMGCR–negative IIM
Number of patients	13	299
Statin exposure prior to onset on IIM	2 (15%)	12/288* (4.2%)
Treatment with IVIG ever	5 (38%)	7 (2.3%)
Female	11 (92%)	182 (61%)

IIM, idiopathic inflammatory myopathy; IVIG, intravenous immunoglobulins; *, information on prior statin use missing for 11 subjects.

Of the 11 statin-naïve anti-HMGCR–positive patients, four were prescribed statins after the diagnosis. Statins had to be withdrawn in three of these four patients due to side effects within 6 months. One patient with statin exposure prior to diagnosis was reintroduced to simvastatin after the initiation of immunosuppressive treatment and did not worsen.

### Clinical Presentation

Among the 13 patients with anti-HMGCR autoantibodies when classified according to the ACR/EULAR criteria from 2017, eight (62%) were as polymyositis and five (38%) as dermatomyositis with typical skin manifestations: Gottron’s sign (30%), Gottron’s papules (15%), heliotrope rash (15%), V-sign (8%), or periungual lesions (23%).

Four anti-HMGCR–positive patients (30%) presented with Raynaud’s phenomenon. Dysphagia occurred in four and ILD was observed in two (15%) patients. Arthritis and mechanic’s hands were present in one patient each (8%).

### Serology Status

Of the five anti-HMGCR–positive patients with dermatomyositis, three were also positive for other MSAs (1 with anti-NXP2 and FHL1, 1 anti-Jo1, 1 anti–TIF1-γ autoantibodies). The patient with anti–TIF1-γ was diagnosed with ovarian cancer 1 month after occurrence of IIM symptoms (**Table 2**).

**Table 2:** Comparison of anti-HMGCR–positive patients with phenotype of polymyositis and dermatomyositis.

	IMNM/Polymyositis	Dermatomyositis	Total anti-HMGCR
Number of patients	8 (62%)	5 (38%)	13
Statin exposure	2 (25%)	0	2 (5%)
Mean age of onset of IIM (years)	54 (range 37–75)	52 (range 28–70)	52
Male	2 (25%)	0	2 (15%)
Female	6 (75%)	5 (100%)	11 (85%)
Patients with multiple MSA	1 (13%)*	3 (60%)**	4 (30%)
Ever treated with IVIG	4 (50%)	1 (20%)	5 (38%)
Strong positivity for anti-HMGCR	5 (63%)	3 (60%)	8 (62%)

IIM, idiopathic inflammatory myopathy; IVIG, intravenous immunoglobulins; IMNM, immune-mediated necrotizing myopathy; MSA, myositis-specific antibodies; *, anti-Jo1; **, anti–TIF1-γ, FHL1 + NXP2, and anti-Jo1.

In the PM/IMNM subgroup, one of eight patients had anti-Jo1 autoantibodies and fulfilled the Connor’s criteria for antisynthetase syndrome (8). There were no anti-HMGCR–positive patients with inclusion body myositis.

Eight patients (62%) had strong positivity of anti-HMGCR antibody, and five patients had weak positivity (38%). Patients with strong positivity had shorter mean time from diagnosis (and initiation of treatment) to sample collection. Dermatomyositis patients were found in both strong- and weak-positive groups. Patients with previous statin exposure had equal representation in both strong and weak positivity for anti-HMGCR antibody subgroups (**Table 2**).

### Pathology Findings

All anti-HMGCR–positive patients had muscle biopsies performed. All biopsies, except for one, revealed histopathological features compatible with immune-mediated necrotizing myopathy: necrotic fibers, fiber size variation, fiber regeneration, and sparse or scattered inflammatory infiltrates. All patients, for whom MHC class I expression was assessed (8 of 13), had upregulation of this molecule in the muscle fibers. MAC staining was performed in only one patient with PM/IMNM and showed presence in the myofibers and capillaries.

### Disease Course

Disease activity was measured longitudinally by the International Myositis Assessment and Clinical Studies Group (IMACS) Core Set Measures. Increased serum levels of CK were observed at the first assessment in nine of 13 (69%) with mean value 29.3 microcat/L (7.54 times upper normal limit, range 1.5–127 microcat/L). Mean MMT8 at the first visit equaled 69/80 (range 55–80). One patient had normal strength at the first assessment and complained of myalgia. This was the patient with the highest serum CK level (27 times the normal upper limit), anti-Jo1 co-positivity, who later gradually improved with treatment (**Supplementary Table**).

At the last follow-up (mean time from the first to the last observation was 161.5 months), all patients had improved after treatment. Mean MMT8 was 76/80 points, mean CK equaled 5.7 microkat/L (1.37 times upper limit), and had normalized in six patients (**Table 3**). Mean dose of steroids at the last follow-up equaled 2.79 mg of prednisone (range 0–10 mg). Most frequently used steroid-sparing agent was azathioprine in eight patients (62%), followed by methotrexate (n = 5, 38%), mycophenolate mofetil (MMF) (n = 4, 30%), rituximab (n = 2, 15%), and cyclophosphamide (n = 2, 15%). High-dose intravenous immunoglobulin (IVIG) was administrated to five (38%) patients. When we compared treatment between anti-HMGCR–positive and anti-HMGCR–negative patients, we observed that 38% of the anti-HMGCR–positive patients had been treated with IVIG. This was significantly more often than in the anti-HMGCR–negative patients (2.3%) (p < 0.005).

**Table 3:** Disease course in anti-HMGCR–positive patients.

	At the diagnosis	At the last follow-up*
Mean CK	29.3 microcat/L (range 1.5–127)	5.7 microcat/L (range 0.9–29.7)
Mean MMT8	69/80 points (range 55–80, median 69/80 points)	73/80 points (range 59–80, median 76/80 points)

CK, creatinine kinase (normal range 0.6–4.7microcat/ml); MMT, manual muscle test; *, mean time from the first assessment was 161.5 months.

Two of the patients with DM required multiple immunosuppressive therapies, whereas the other two had good response and were stable without treatment at the last visit. One patient with mild muscle weakness and normal CK was treated without systemic steroids but with hydroxychloroquine and topical therapy with good results (**Supplementary Table**).

From the two patients with statin exposure history, one had refractory disease requiring use of rituximab, and another was managed primarily with methotrexate and then low-dose prednisone at last follow-up.

At the last assessment, five patients were in remission (including three with drug-free remission), five had chronic active disease, and two had low disease activity. One patient had positive anti–TIF1-γ autoantibodies with a clinical phenotype of dermatomyositis, but their muscle biopsy had clear pathological features of IMNM. This patient died due to ovarian cancer (**Supplementary Table**).

### Genetic Assessment

We did not see significant enrichment of any HLA-DRB1 genotype/allele in analyzed group of anti-HMGCR–positive patients. None were found with HLA-DRB1*08:03 nor HLA-DRB1*11:01 (9).

## Discussion

In this cross-sectional study of a European cohort of patients with IIM, we recorded a frequency of anti-HMGCR autoantibodies of 4.3%. Two of the 13 anti-HMGCR–positive patients had previously been treated with statins.

In a previous study from China, 405 patients with IIM were examined and anti-HMGCR autoantibodies were present in 22 individuals (5.4%), whereas previous statin use was ascertained in only four (16%) (10), which is similar to our results. In another cohort of 227 Chinese IIM patients, 21 were found positive for anti-HMGCR and nine had clinical features of dermatomyositis, but the muscle biopsy features resembled IMNM, and none had typical histopathological DM findings (11).

In a Japanese cohort of 460 patients with IIM, anti-HMGCR antibodies were detected in 12% (10). Muscle biopsies were most often consistent with IMNM. Chronic progression of muscle weakness over 12 months, which mimicked muscular dystrophy, was also observed. Most patients required multiple immunosuppressants. Exposure to statins was confirmed in 18% of these anti-HMGCR–positive subjects (10).

One European study exclusively included patients with IMNM and anti-HMGCR autoantibodies; nevertheless, 57% were found with skin lesions attributable to the disease (**Table 4**) (12).

**Table 4:** Comparison of studies screening large IIM cohorts for anti-HMGCR autoantibodies.

	Ge et al. (11)	Watanabe et al. (10)	Williams et al. (12)	Current study
Population	405 patients (Bohan and Peter diagnostic criteria from 1975)	387 patients (exclusion of non-IIM and IBM patients)	23 IMNM anti-HMGCR–positive patients	312 patients (ACR/EULAR classification criteria from 2017)
Anti-HMGCR autoantibodies assessment method	ELISA	ELISA	Chemiluminescence	ELISA, IP-IVTT
Anti-HMGCR–positive patients	22 (5.4%)	47 (12%)	23 (100%)	13 (4.3%)
Dermatomyositis phenotype	8 (36%)	2 (4%)	13 (57%)	5 (38%)
Females	16 (73%)	31 (69%)	8 (35%)	11 (92,3%)
Previous exposure to statins	3/20 (15%)	8 (18%)	20 (87%)	2 (15%)
Dysphagia	11 (50%)	20 (44%)	–	4 (31%)
Elevated CK at the diagnosis	18/21 (85.6%)	42 (93%)	100%	9 (69%)
IMNM features in muscle biopsy	8/12 (67%)	–	19/23 (83%)	12 (92%)
Co-occurrence of other MSA	3 (14%)	0	–	4 (31%)

IIM, idiopathic inflammatory myopathies; IBM, inclusion body myositis; CK, creatinine kinase; IMNM, immune-mediated necrotizing myopathy; MSA, myositis-specific autoantibodies; ELISA, enzyme-linked immunosorbent assay; IP-IVTT, immunoprecipitation of in vitro transcribed and translated protein.

To our knowledge, this is the first study that performed testing for anti-HMGCR autoantibodies in a large European, mainly Caucasian cohort of IIM patients. As a result, a wide spectrum of symptoms among patients positive for anti-HMGCR was observed. In our study, five patients (38%) with anti-HMGCR autoantibodies had skin rash attributable to dermatomyositis. One of them had muscle pathology findings consistent with DM, whereas the other four predominantly had features of IMNM on muscle biopsy. Two patients had accompanying perifascicular atrophy, which is a known histopathological feature of DM. Dermatomyositis cases with anti-HMGCR autoantibodies have been anecdotally reported previously; one study even suggested presence of distinctive skin lesions (13). Here, we present a group of patients with both clinical and histopathological features of dermatomyositis associated with anti-HMGCR autoantibodies.

Some of our patients had a mild disease course, three of them had normal CK at the onset, and one patient had normal muscle strength (**Supplementary Table**). Five patients were in remission (including three with drug-free remission) at the last follow-up. One patient with anti-HMGCR autoantibodies had muscle weakness resembling limb-girdle muscular dystrophy. This similarity has previously been described (14). In this particular patient, this ambiguity caused delay in diagnosis for many years with suboptimal treatment as a result.

Because serology was performed retrospectively on archived sera, the patients in our cohort were managed based on their clinical phenotype and the treatment approach had not been influenced by the awareness of anti-HMGCR positivity. Given this fact, it is interesting that almost half of the anti-HMGCR–positive patients were treated with IVIG, especially at a center that rarely uses this medication. This supports the hypothesis that routinely adding anti-HMGCR to baseline diagnostic assessment could be advantageous in predicting course of disease and adjusting optimal treatment, even if the phenotype is not always consistent. Histopathology features of IMNM, namely, necrotic, regenerating myofibers, fiber size variation, or scattered inflammatory cells, were present in almost all specimens of the anti-HMGCR–positive subjects, including those with dermatomyositis. Only one dermatomyositis patient had pathology findings strongly indicating DM. A limitation of the study is that most of the samples lacked MAC staining, none was stained for p62, which would be a great asset, but it was not a routine procedure at the time when most of the samples were stained.

An interesting observation of our study is presence of other myositis-specific antibodies (MSA) in 30% of patients, although they were not confirmed by other laboratory methods. Furthermore, the line blot results of patients positive for anti–TIF1-γ, anti-NXP2, and anti-Jo1 corresponded to typical clinical presentation. The seropositivity for anti-HMGCR can have a false positive rate of up to 0.7%, prompting our confirmation with IP-IVTT. Again, this suggests that adding anti-HMGCR to routine assessment can be helpful in the diagnostic workup even when other MSAs are present. Of note, 70% of the anti-HMGCR–positive patients would have remained seronegative, if the anti-HMGCR antibodies were not assessed.

The number of patients with history of statin use prior to the onset of myositis (two cases) is smaller than in previous reports (10, 11, 15). However, this likely speaks to selection bias between IMNM and anti-HMGCR–positive DM patients. It is also worth mentioning that prescription of statins during the time of serum collection (1988–2014) was not common in Sweden, as non-pharmacologic interventions have been regarded as primary treatment. In our observation of the latest patients with anti-HMGCR autoantibodies from the same site, the proportion of patients exposed to statins has increased. This study is unique as we analyzed archived sera from before the wider introduction of statins.

Statins are not the only identified HMGCR inhibitors; other known sources are red yeast rice and certain mushrooms species (e.g., *Pleurotus* spp. and *Agaricus bisporus*), many probably remain yet to be discovered.

With this study, we propose to include anti-HMGCR autoantibody testing in the IIM diagnostic routine, applying to patients with phenotype of polymyositis, dermatomyositis, and, perhaps, some cases of asymptomatic hyper-CK-emia and myalgia (e.g., unclear cases resembling muscle dystrophies).

## Conclusions

Patients with IIM related to anti-HMGCR autoantibodies may present with a wide range of symptoms. A minority in our cohort had previous statin exposure, which suggests a different pathogenesis than drug-related. This study indicates that screening for anti-HMGCR autoantibodies in patients with suspected IIM is justified and could be helpful in the diagnostic workup not only in individuals with previous statin exposure. Longitudinal follow-up of large cohorts will contribute to understanding the prognostic value of anti-HMGCR autoantibodies in a broad clinical spectrum of disease.

## Data Availability Statement

The original contributions presented in the study are included in the article/**Supplementary Material**. Further inquiries can be directed to the corresponding author.

## Ethics Statement

The studies involving human participants were reviewed and approved by Regional Ethics Committee, Stockholm, Sweden, project number 2005/792-31, 2008/449-32, 2011/1374-32. The patients/participants provided their written informed consent to participate in this study.

## Author Contributions

Conception and design: MD, PS, SB. Data collection: MD, PS, SB. Analysis and interpretation of data: MD, SB, PS. Evaluation of muscle biopsies: IN, OD. Writing of the manuscript, revising it critically: MD, PS, SB, IN, OD. IN: Evaluation of the muscle biopsy samples, analyzing of data, and participating in manuscript preparation. MD: supervision and coordination of work and participating in manuscript preparation. All authors contributed to the article and approved the submitted version.

## Funding

Funding for this study came from the Swedish Research Council, the Swedish Rheumatism Association, King Gustaf V 80 Year Foundation, Stockholm County Council (ALF project).

## Conflict of Interest

The authors declare that the research was conducted in the absence of any commercial or financial relationships that could be construed as a potential conflict of interest.

## Publisher’s Note

All claims expressed in this article are solely those of the authors and do not necessarily represent those of their affiliated organizations, or those of the publisher, the editors and the reviewers. Any product that may be evaluated in this article, or claim that may be made by its manufacturer, is not guaranteed or endorsed by the publisher.
