# Supplementary material for: Screening for Anti-HMGCR Antibodies in a Large Single Myositis Center Reveals Infrequent Exposure to Statins and Diversiform Presentation of the Disease
Source: Front Immunol. 2022 May 4;13:866701. doi: 10.3389/fimmu.2022.866701 (PMC9114810; doi:10.3389/fimmu.2022.866701)
Supplement: Supplementary file 1 [file Table_1.pdf]

doi: 10.3389/fimmu.2022.866701

Screening for anti-HMGCR Antibodies in a Large Single Myositis Center Reveals Infrequent Exposure to Statins and Diversiform Presentation of the Disease

Supplementary table.

	Age of onset	Gender	Previous statin exposure	Serology besides anti-HMGCR	Anti-HMGCR positivity	Time from treatment initiation to sampling (months)	Phenotype	Extra-muscular involvement	CK at the first assessment (times upper normal range)	MMT8 at the first assessment [0-80]	Main biopsy findings	Treatment ever	Treatment at the last assessment	Outcome
**1.**	75	Male	Yes	Ro60, Ro52	Weak	0	PM	H	2	78	NF, RGF, FSV, PMA, EI, MHC	GCS, MTX	GCS 10mg	Chronic active
**2.**	70	Female	No	-	Strong	0	DM	ILD, S, R, D	25.3	55	NF, RGF, FSV, MHC	GCS, AZA, CYC, RTX	AZA 50mg, GCS 2,5mg	Remission
**3.**	70	Female	Yes	-	Strong	0	PM	-	25.2	61	NF, RGF, FSV, MHC	GCS, AZA, RTX, MMF, IVIG	GCS 10mg	Chronic active
**4.**	64	Female	No	NXP2, FHL1	Weak	0	DM	S	Normal	77	NF, RGF, FSV, PFA, MHC	GCS, AZA, MMF	MMF 2g, GCS 2,5mg	Remission
**5.**	58	Male	No	Jo-1, SSA	Strong	0	PM	-	27	68	NF, FGF, SI, EI, PI, MAC, MHC	GCS, MTX	none	Drug free remission
**6.**	54	Female	No	-	Strong	0	PM	D	2.5	67	NF, RGF, SI, FSV	GCS, MTX, AZA, CYC, IVIG, MMF	MMF, IVIG 2g/kg/month, GCS 2,5mg	Chronic active
**7.**	52	Female	No	TIF1-γ	Strong	16	DM	S, D	Unknown	-	NF, RGF, MHC	GCS, MTX	Death due to malignancy	Death due to malignancy
**8.**	51	Female	No	Jo-1, Ro60, Ro52	Weak	118	DM	ILD, S, R, A	Normal	71	FSV	GCS, AZA	none	Drug free remission
**9.**	48	Female	No	-	Weak	86	PM	-	1.2	59	NF, FSV	GCS, AZA	GCS 6.25mg	Chronic active
**10.**	47	Female	No	-	Strong	73	PM	-	2.2	-	NF, RGF, FSV, PFA	GCS, MTX, AZA, IVIG	none	Low disease activity
**11.**	47	Female	No	-	Weak	11	PM	H, R, D	1.7	69	RGF, SI, PI, MHC	GCS, MMF, IVIG	MMF 2g, GCS 5mg	Chronic active
**12.**	37	Female	No	-	Strong	0	PM	S	1.8	76	FSV	GCS, AZA, IVIG	none	Low disease activity
**13.**	28	Female	No	ACA	Strong	0	DM	S, R	Normal	80	SI, PI, PFA, MHC	HHQ	none	Drug free remission

CK – creatine kinase, MMT8 – manual muscle test of 8 muscle groups, PM – polymyositis (including IMNM), DM – dermatomyositis, GCS – glucocorticoids (prednisone), AZA – azathioprine, MMF – mycophenolate mofetil, MTX – methotrexate, IVIG – intravenous immunoglobulins, RTX – rituximab, HHQ – hydroxychloroquine, ACA – anti-centromere antibody, ILD – interstitial lung disease, S – skin lesions, R – Raynaud's phenomenon, A – arthritis, D – dysphagia, NF – necrotic fibers, RGF – regenerating fibers, FSV – fiber size variation, PMA – perimysial atrophy, EI – endomysial inflammatory infiltrates, PI – perimysial inflammatory infiltrates, SI – scattered inflammatory infiltrates, MAC – membrane attack complex, MHC – major histocompatibility complex class I upregulation
